# Maternal Choline Supplementation as a Potential Therapy for Down Syndrome: Assessment of Effects Throughout the Lifespan

**DOI:** 10.3389/fnagi.2021.723046

**Published:** 2021-10-06

**Authors:** Brian E. Powers, Ramon Velazquez, Myla S. Strawderman, Stephen D. Ginsberg, Elliott J. Mufson, Barbara J. Strupp

**Affiliations:** ^1^Division of Nutritional Sciences, Cornell University, Ithaca, NY, United States; ^2^Edward Hines Jr. VA Hospital, Hines, IL, United States; ^3^Department of Psychology, Cornell University, Ithaca, NY, United States; ^4^Arizona State University-Banner Neurodegenerative Disease Research Center, Biodesign Institute, Arizona State University, Tempe, AZ, United States; ^5^School of Life Sciences, Arizona State University, Tempe, AZ, United States; ^6^Center for Dementia Research, Nathan Kline Institute, Orangeburg, NY, United States; ^7^Department of Psychiatry, New York University Grossman School of Medicine, New York, NY, United States; ^8^Department Neuroscience and Physiology, New York University Grossman School of Medicine, New York, NY, United States; ^9^New York University Neuroscience Institute, New York University Grossman School of Medicine, New York, NY, United States; ^10^Departments of Translational Neuroscience and Neurology, Barrow Neurological Institute, St. Joseph's Medical Center, Phoenix, AZ, United States

**Keywords:** Down syndrome, maternal choline supplementation, aging, learning, attention, septohippocampal circuit

## Abstract

Maternal choline supplementation (MCS) has emerged as a promising therapy to lessen the cognitive and affective dysfunction associated with Down syndrome (DS). Choline is an essential nutrient, especially important during pregnancy due to its wide-ranging ontogenetic roles. Using the Ts65Dn mouse model of DS, our group has demonstrated that supplementing the maternal diet with additional choline (4-5 × standard levels) during pregnancy and lactation improves spatial cognition, attention, and emotion regulation in the adult offspring. The behavioral benefits were associated with a rescue of septohippocampal circuit atrophy. These results have been replicated across a series of independent studies, although the magnitude of the cognitive benefit has varied. We hypothesized that this was due, at least in part, to differences in the age of the subjects at the time of testing. Here, we present new data that compares the effects of MCS on the attentional function of adult Ts65Dn offspring, which began testing at two different ages (6 vs. 12 months of age). These data replicate and extend the results of our previous reports, showing a clear pattern indicating that MCS has beneficial effects in Ts65Dn offspring throughout life, but that the magnitude of the benefit (relative to non-supplemented offspring) diminishes with aging, possibly because of the onset of Alzheimer's disease-like neuropathology. In light of growing evidence that increased maternal choline intake during pregnancy is beneficial to the cognitive and affective functioning of all offspring (e.g., neurotypical and DS), the addition of this nutrient to a prenatal vitamin regimen would be predicted to have population-wide benefits and provide early intervention for fetuses with DS, notably including babies born to mothers unaware that they are carrying a fetus with DS.

## Introduction

Down syndrome (DS) is the most common genetic cause of intellectual disability, characterized by deficits in language comprehension and production, as well as impairments of learning, memory, and various executive functions (e.g., planning, inhibitory control, and attention) (Chapman and Hesketh, 2000; Rachidi and Lopes, 2010). In addition, nearly all individuals with DS develop the neuropathological changes associated with Alzheimer's disease (AD) by the third to fourth decade of life, including senile plaques, neurofibrillary tangles, and degeneration of basal forebrain cholinergic neurons (BFCNs) (Wisniewski et al., 1985a,b; Mann et al., 1986; Lai and Williams, 1989; Leverenz and Raskind, 1998; Hartley et al., 2015). There are currently no effective therapies to prevent or ameliorate the cognitive impairment and brain pathology associated with DS, so the identification of interventions is of paramount importance.

Several mouse models have been generated that recapitulate the hallmark phenotype of humans with DS, providing translational tools to elucidate the pathogenic processes associated with this disorder and the testing of potential therapies. The Ts65Dn mouse model is well-characterized and replicates key aspects of human DS neuropathology and associated behavioral deficits (Davisson et al., 1990; Reeves et al., 1995; Holtzman et al., 1996; Hyde and Crnic, 2001; Hyde et al., 2001a,b; Bimonte-Nelson et al., 2003; Hunter et al., 2003). Notably, like humans with DS, Ts65Dn mice exhibit impairments in hippocampal-dependent learning and memory, attentional dysfunction, hyperactivity, and heightened emotionality (Granholm et al., 2000; Hyde et al., 2001b; Driscoll et al., 2004; Moon et al., 2010; Velazquez et al., 2013; Ash et al., 2014; Powers et al., 2016, 2017, 2018). Also similar to humans with DS, Ts65Dn mice are born with intact BFCNs, and projections from these neurons to the hippocampus (septohippocampal circuit) and neocortex (basocortical circuit) show normal organization. But, similar to the early-onset degeneration of these projection systems seen in adult humans with DS, Ts65Dn mice display progressive BFCN atrophy beginning at about 6 months of age (MO) (Holtzman et al., 1996; Granholm et al., 2000; Cooper et al., 2001), coincident with a progressive decline in cognitive functioning (Hyde and Crnic, 2001).

Our group has utilized the Ts65Dn mouse model to test the hypothesis that maternal choline supplementation (MCS) improves cognitive and affective functioning in DS, as well as protects against age-related degeneration of BFCNs that give rise to the septohippocampal and basocortical connectomes. Choline is an essential nutrient, and its availability during pregnancy is a key factor in fetal neurodevelopment (Zeisel, 2006). Choline is a precursor for phosphatidylcholine and sphingomyelin, two critical components of neuronal and non-neuronal lipid membranes, and it is essential for the biosynthesis of acetylcholine, a neurotransmitter that regulates neuronal proliferation, differentiation, migration, plasticity, and synapse formation (Lauder and Schambra, 1999; Abreu-Villaça et al., 2011; Bernhard et al., 2019). Choline is also the primary dietary source of methyl groups, thereby contributing to the epigenetic regulation of gene expression *via* DNA and histone methylation (Kovacheva et al., 2009; Blusztajn and Mellott, 2012; Jiang et al., 2012). Because of the wide-ranging ontogenetic roles of choline, dietary demand is markedly increased during pregnancy, when there is a pronounced depletion of maternal choline stores throughout gestation (Yan et al., 2012; Jiang et al., 2014). Importantly, numerous studies using normal rodents have demonstrated that increasing maternal intake of choline during critical periods of early development has lasting beneficial effects on cognitive and affective functioning of offspring. Specifically, supplementing the maternal diet with additional choline during pregnancy (approximately four times higher than standard lab chow) has been shown to improve memory, spatial cognition, and attentional function of offspring (Meck and Williams, 2003; McCann et al., 2006), and also emotion regulation (Cheng et al., 2008)—all domains which are adversely affected in DS.

Over the past 10 years, we have demonstrated that supplementing the diet of Ts65Dn dams during pregnancy and lactation with additional choline is beneficial to trisomic offspring (Moon et al., 2010; Velazquez et al., 2013; Ash et al., 2014; Powers et al., 2017). Specifically, supplemented trisomic mice (vs. non-supplemented counterparts) exhibited significant improvements in spatial cognition, attention, and emotion regulation (Moon et al., 2010; Velazquez et al., 2013; Ash et al., 2014). These same studies also revealed that MCS normalizes adult hippocampal neurogenesis in the trisomic offspring and prevents degeneration of BFCNs of the medial septum. Moreover, the behavioral indices of spatial cognition correlated significantly with both hippocampal neurogenesis and medial septal cholinergic neuron density, suggesting that they contribute to the functional benefits. We have also shown that MCS normalizes the expression of genes associated with synaptic plasticity, calcium signaling, and neurodegeneration in the septohippocampal circuit (Alldred et al., 2018, 2019), providing plausible underlying mechanisms of neurodegeneration.

Although benefits of MCS for Ts65Dn mice have been replicated across several studies (Moon et al., 2010; Velazquez et al., 2013; Ash et al., 2014; Powers et al., 2017), it is notable that the magnitude of improvement in attentional function has varied across studies, for reasons that we are only now beginning to understand. Specifically, a robust benefit of MCS was observed in trisomic offspring when attentional function was assessed between 6 and 10 MO (Moon et al., 2010). By contrast, when testing occurred between 12 and 17 MO, a benefit of MCS for trisomic offspring was seen for spatial cognition, hippocampal neurogenesis, and neuroprotection of BFCNs, but was less pronounced for attention (Velazquez et al., 2013; Ash et al., 2014; Powers et al., 2017). The factors underlying these discrepancies were unclear since the housing facility, testing equipment, personnel, and age at which the attentional studies were performed differed.

Here, we present new data from two investigations, which strongly indicate that the age of subjects at the time of testing was likely responsible for these differences in outcome. In the first study (Study 1 herein), we initiated behavioral testing of the offspring at 12 MO, so that gene expression and other neurologic endpoints could be compared and contrasted with our prior neurological findings (Velazquez et al., 2013; Ash et al., 2014; Powers et al., 2017). Here, we did not observe significant benefits of MCS on attentional function; these data were similar to the modest effects of MCS that we reported previously (Powers et al., 2017), when the offspring were tested at this older age. These findings led to the hypothesis that the reduced magnitude of the attentional benefit was due to the older age of subjects at the time of testing. To test this hypothesis, we conducted another study, which initiated attention testing at 6 MO (Study 2 herein). As observed in our original report on MCS in Ts65Dn mice of this same age (Moon et al., 2010), we found robust improvement in attentional function in the choline-supplemented trisomic offspring, relative to non-supplemented trisomics. Together these four studies lend support to our hypothesis that attentional benefits of MCS occur during early- and mid-life in trisomic offspring, but diminish with aging.

## Methods (Studies 1 and 2)

The methods for both studies are identical other than the age at which behavioral testing was initiated. Thus, the following methods pertain to both studies, unless otherwise noted.

### Subjects

Breeder pairs (female Ts65Dn and male C57Bl/6J Eicher × C3H/HeSnJ F1 mice) were purchased from Jackson Laboratories (Bar Harbor, Maine) and mated at Cornell University, Ithaca, New York, USA. All dams are trisomic but give birth to both trisomic and normal disomic (2N) mice. Upon arrival, breeder pairs were randomly assigned to receive either a standard purified rodent diet (containing choline) or one supplemented with additional choline. The control diet was AIN-76A purified rodent diet containing 1.1 g/kg choline chloride, whereas the choline-supplemented diet was the same (AIN-76A purified rodent diet) but containing 5.0 g/kg choline chloride (Dyets Inc., Bethlehem, Pennsylvania, USA). The control diet supplies adequate choline as recommended by the National Research Council, Nutrient Requirements of Laboratory Animals, Fourth Revised Edition [National Research Council (US) Subcommittee on Laboratory Animal Nutrition, 1995]. The choline-supplemented diet provided ~4.5 times the concentration of choline, within the range of dietary variation observed in humans (Detopoulou et al., 2008). Breeder pairs were provided *ad libitum* access to water and their assigned diets, and remained on this diet throughout the prenatal and postnatal periods. Standard cages contained paper bedding and several objects (e.g., plastic igloo, *t*-tube, plastic-gel bone, and cotton square). Mice were maintained on a 12-h light-dark cycle under temperature- and humidity-controlled conditions.

Offspring were weaned on postnatal day (PND) 21, and all subjects were provided *ad libitum* access to water and the control diet. Thus, choline supplementation was provided only maternally, starting before conception and ending on PND 21. Ear punch tissue was sent to Jackson Laboratories for genotyping by quantitative polymerase chain reaction for the detection of the extra chromosomal segment and determination of *Pde6Brd1* homozygosity, a recessive mutation leading to retinal degeneration (Bowes et al., 1993). *Pde6Brd1* homozygous mice were excluded from the study.

For each study, we used male mice (*n* = 16) comprising four groups: (i) disomic offspring of dams on the control diet (**2N**); (ii) Ts65Dn offspring of dams on the control diet (**Ts**); (iii) disomic offspring of dams on the choline-supplemented diet (**2N+**); and (iv) Ts65Dn offspring of dams on the choline-supplemented diet (**Ts+**). There was some attrition due to animal health concerns or the failure to perform the basic training task. The final subject numbers included in statistical analyses in Study 1 were: 2N (*n* = 14), 2N+ (*n* = 16), Ts (*n* = 15), and Ts+ (*n* = 15). In Study 2: 2N (*n* = 13), 2N+ (*n* = 16), Ts (*n* = 15), and Ts+ (*n* = 13). One month prior to behavioral testing, mice were moved to a room with a 13:11-h reversed light-dark cycle (lights off at 8:00 a.m., lights on at 9:00 p.m.), and were singly housed to prevent fighting, which can occur when group-housed male mice of this strain are returned to the home cage following behavioral test sessions. Mice were placed on a food restriction regimen to ensure motivation for food rewards during testing. Target weights were calculated at ~85% of their *ad libitum* weight.

All protocols were approved by the Institutional Animal Care and Use Committee of Cornell University and conform to the National Institutes of Health Guide for the Care and Use of Laboratory Animals.

### Behavioral Testing

Study 1 initiated testing at ~12 MO, whereas Study 2 began at 6 MO. For simplicity, the mice in Study 1 will be referred to as “aged mice” and in Study 2 as “young mice.”

Subjects were tested individually in 1 of 8 PC-controlled operant chambers. The chambers were manufactured in-house, with the body constructed by Ithaca Plastics Inc. (Ithaca, New York, USA) as described previously (Powers et al., 2017). One wall contained a retractable door, controlling access to a dipper (ENV0302M, MED Associates, East Fairfield, Vermont, USA) that dispensed a liquid food reward (Liquefied AIN-76A; Bio-Serv, Frenchtown, New Jersey, USA). The opposite wall contained five nosepoke response ports, each with a green 4-mA LED embedded on the back surface. Mice were pseudorandomly assigned to chambers so that an equal number of mice from each group was tested in each chamber.

Subjects were tested once per day, 6 days per week, and each session lasted 30 min or 70 trials, whichever came first. Mice were weighed and placed into the chambers by experimenters blinded to genotype and diet. After the test session, each subject was returned to the homecage and fed 30 min later. Chambers were thoroughly cleaned after each session using Odormute (R.C. Steele Co, Brockport, New York, USA). Subjects progressed sequentially through a series of training and attention tasks. The total duration of testing spanned a period of 5 months.

A series of training tasks familiarized the subjects with the test chambers and the sequence of responses necessary to complete a trial for the attention tasks (see Powers et al., 2017). Mice then began a five-choice visual discrimination task. On each trial, one of the five nosepoke response port LEDs was illuminated and remained illuminated until the mouse made a nosepoke into one of the ports or until 32 s elapsed. The location of the visual cue was pseudorandomized across trials so that the number of cue presentations in each port was balanced for each daily session. The subject was rewarded with the liquid food for making a nosepoke into the illuminated (i.e., correct) port. Following an error, or if the animal failed to nosepoke, the dipper door remained closed and a 5-s time-out period was imposed, signaled by the illumination of a 3W house light on the ceiling of the chamber. The interval between trials was 5 s. Each subject remained on this initial visual discrimination task until it reached a criterion of 80% correct for two of three consecutive sessions. Subjects required ~600–700 trials to reach the criterion, which is consistent with the results of our prior experiments (Moon et al., 2010; Powers et al., 2016, 2017).

Subjects were then tested for eight sessions on Attention Task 1. This task was the same as the initial visual discrimination task, except that the duration of cue illumination was shortened to 1 second. Subjects were subsequently tested for 18 sessions on Attention Task 2, which imposed a variable delay of 0, 2, or 4 s between trial initiation and cue illumination. The pre-cue delays were presented pseudorandomly so that the number of presentations of each combination of pre-cue delay and response port (1–5) was balanced across each session. If a response was made prior to cue onset (i.e., premature), the trial was terminated, and no cue was presented. Finally, subjects were tested for 10 sessions on Attention Task 3, which likewise imposed a variable pre-cue delay of 0, 2, or 4 s, but also had a variable cue duration of either 0.8, 1.0, or 1.4 seconds. Attention Tasks were each initiated on the day following completion of the prior task.

Several types of errors were possible: (i) making a nosepoke into any port prior to cue onset (premature), (ii) responding to a non-illuminated port (inaccurate), and (iii) failing to respond to any port within 32 s of cue onset (omission).

### Statistical Analysis

Statistical analyses were performed using the Statistical Analysis System (Version 9.3; SAS Institute, Cary, North Carolina, USA). Data were analyzed using PROC GLM and PROC GLIMMIX. PROC GLIMMIX is a generalized linear mixed models procedure for conducting repeated measures analyses. Fixed factors for all tasks included genotype and maternal diet. Pre-cue delay and stimulus duration were also included for tasks in which these parameters varied. For Attention Task 2, session block (blocks of three daily sessions) was also included so that we could assess the rate of acquisition as the mice learned the new task rule (i.e., a variable delay was imposed between trial onset and cue illumination). Dependent measures included trials to criterion, errors to criterion, percentage of correct responses, percentage of inaccurate responses, percentage of omission errors, and percentage of premature responses. Planned comparisons were made between Ts and Ts+ mice and between 2N and 2N+ mice. The alpha level was set at *p* < 0.05 for all analyses.

## Results

There were no significant main effects of genotype or maternal diet on the number of trials to criterion or errors to criterion on the initial five-choice visual discrimination task. Thus, all subjects began Attention Task 1 with a similar mastery of the basic skills and associations required to perform these tasks.

### Attention Task 1 (1 s Cue Duration)

#### Study 1 (Aged Mice)

**Figure 1:**
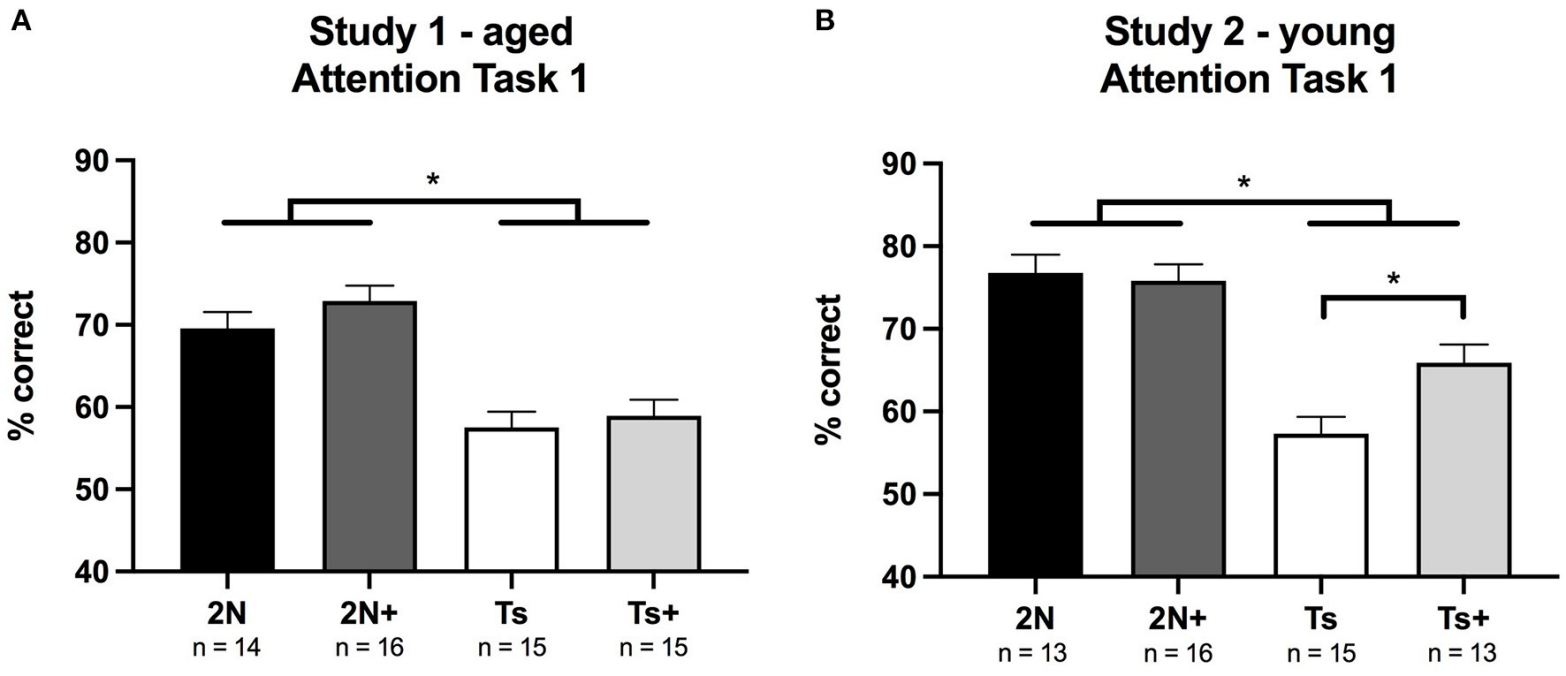
Percentage of correct responses on Attention Task 1 (1 s cue duration) in Study 1 (Panel **A**) and Study 2 (Panel **B**). **(A)** In Study 1 (aged mice), a significant effect of genotype was detected with 2N mice performing better than Ts mice. **(B)** In Study 2 (young mice), a significant effect of genotype was seen as well as a significant genotype by maternal diet interaction. The supplemented trisomic mice performed better than the non-supplemented trisomic mice. *p < 0.05.

In Study 1 (aged mice), analysis of percentage correct for Attention Task 1 revealed a significant main effect of genotype [*F*_(1, 56)_ = 44.56, *p* < 0.0001] indicating that Ts65Dn mice performed more poorly than 2N mice. There was no significant main effect of maternal diet, nor a significant genotype by maternal diet interaction (Figure 1A). Analysis of error types revealed a significant main effect of genotype on the percentage of omissions [*F*_(1, 56)_ = 21.52, *p* < 0.0001] indicating that the poor performance of the Ts65Dn mice was driven by an increase in these errors (Table 1). Note that this was the first task in which the cue duration was relatively brief, and the trisomic mice were impaired in their ability to detect and respond to these brief cues.

**Table 1:** Average percentage of each error type for each group for each attention task.

		**Study 1**				**Study 2**			
		**2N**	**2N+**	**Ts**	**Ts+**	**2N**	**2N+**	**Ts**	**Ts+**
Attention Task 1	% inaccurate	18.51 ± 1.91	16.08 ± 1.62	20.49 ± 2.31	18.19 ± 2.11	14.53 ± 1.68	14.24 ± 1.52	18.83 ± 1.71	13.68 ± 1.72
	% omission	6.83 ± 1.24	7.03 ± 1.38	13.81 ± 1.66	15.62 ± 1.73	5.20 ± 1.51	6.82 ± 1.64	11.83 ± 1.57	12.72 ± 1.58
Attention Task 2	% premature	28.33 ± 3.18	26.83 ± 3.21	30.83 ± 3.12	26.33 ± 2.95	27.29 ± 3.24	25.77 ± 2.93	37.48 ± 3.02	28.76 ± 3.25
	% inaccurate	8.48 ± 0.79	6.41 ± 0.82	9.84 ± 0.79	9.82 ± 0.79	6.72 ± 0.91	6.74 ± 0.83	9.51 ± 0.85	8.74 ± 0.92
	% omission	12.05 ± 2.33	13.08 ± 1.89	18.17 ± 1.93	20.67 ± 2.23	8.58 ± 1.61	9.09 ± 1.73	17.31 ± 1.26	14.62 ± 1.78
Attention Task 3	% premature	10.12 ± 1.70	9.21 ± 1.39	13.27 ± 1.38	11.51 ± 1.51	6.63 ± 1.20	8.54 ± 1.08	15.41 ± 1.11	10.20 ± 1.19
	% inaccurate	6.30 ± 1.14	6.26 ± 1.01	7.99 ± 0.96	7.72 ± 1.07	5.19 ± 1.02	5.75 ± 0.93	8.07 ± 0.92	7.97 ± 0.10
	% omission	11.16 ± 2.70	12.26 ± 2.21	20.11 ± 2.19	20.74 ± 2.41	9.28 ± 1.76	8.62 ± 1.59	18.80 ± 1.64	15.38 ± 1.76

#### Study 2 (Young Mice)

In Study 2 (young mice), analysis of percentage correct for Attention Task 1 revealed a significant main effect of genotype [*F*_(1, 53)_ = 50.62, *p* < 0.0001], with Ts65Dn mice again performing more poorly than 2N counterparts. There was also a significant genotype by maternal diet interaction [*F*_(1, 53)_ = 5.12, *p* < 0.02]. Planned comparisons revealed that choline-supplemented Ts65Dn mice performed significantly better than their non-supplemented counterparts (*p* < 0.01; Figure 1B).

Analysis of error types revealed significant main effects of genotype on the percentage of omissions [*F*_(1, 53)_ = 24.58, *p* < 0.0001] and inaccurate responses [*F*_(1, 53)_ = 20.66, *p* < 0.001], indicating that trisomic mice committed a higher percentage of both types of errors (Table 1). Planned comparisons indicated that improvement in the choline-supplemented Ts65Dn mice for percentage correct was due to fewer inaccurate responses relative to the non-supplemented Ts65Dn mice (*p* < 0.01). Premature responses were not analyzed for this task because there were no pre-cue delays.

### Attention Task 2 (0, 2, or 4 s Variable Pre-cue Delay; 1 s Cue Duration)

In both Study 1 and Study 2, pre-cue delay had a significant effect on performance for all groups of mice; percent correct decreased as the duration of the pre-cue delay increased (*p* < 0.0001). The pre-cue delay also had significant effects on each error type (premature, inaccurate, and omission) for all groups in both studies (*p* < 0.0001).

#### Study 1 (Aged Mice)

**Figure 2:**
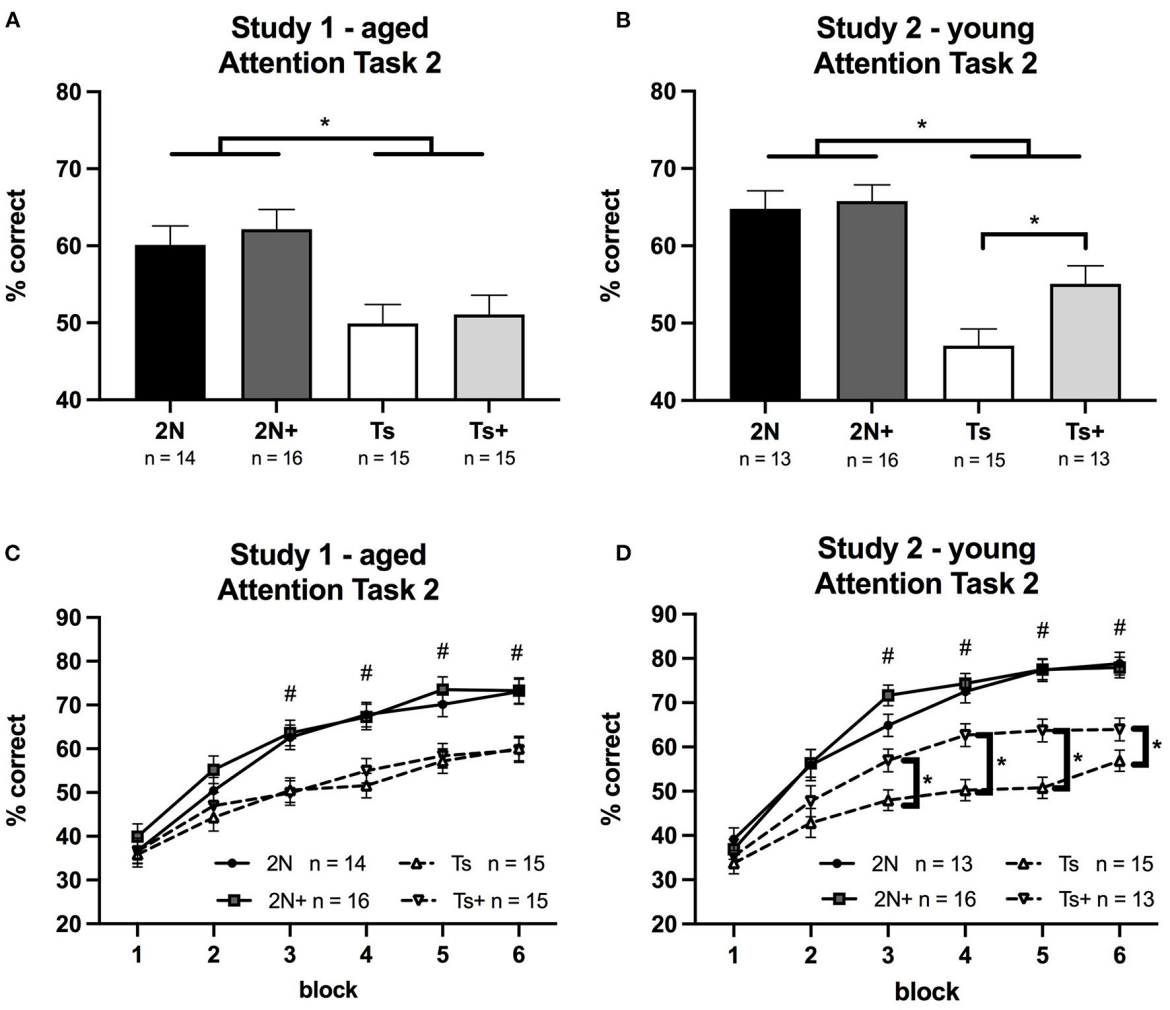
Percentage of correct responses on Attention Task 2 (1 s cue duration; 0, 2, or 4 s pre-cue delay) in Study 1 and Study 2. Panels **(A,B)** depict percent correct averaged across the entire task, whereas Panels **(C,D)** depict performance as a function of testing block. **(A)** In Study 1 (aged mice), a significant effect of genotype was detected, reflecting the superiority of the 2N vs. trisomic mice. **(B)** In Study 2 (young mice), a significant effect of genotype was detected as well as a significant effect of maternal diet. Planned comparisons revealed that within the Ts65Dn mice, there was a significant effect of maternal diet (*p* < 0.02) with the supplemented trisomic mice performing better than non-supplemented trisomic mice. **(C)** In Study 1 (aged mice), there was a significant genotype by block interaction with 2N mice performing better than Ts mice during blocks 3–6. **(D)** In Study 2 (young mice), there was a significant genotype by block interaction and a significant maternal diet by block interaction. ^#^The 2N and 2N+ groups were both significantly different from each Ts group (*p* < 0.05). *The supplemented trisomic mice performed better than the non-supplemented trisomic mice during blocks 3–6 (*p* < 0.05).

In Study 1 (aged mice), the analysis of percentage correct for Attention Task 2 revealed a significant main effect of genotype [*F*_(1, 56)_ = 18.03, *p* < 0.0001] reflecting significant impairment of the Ts65Dn mice relative to 2N mice (Figure 2A). This analysis also revealed a significant genotype by block interaction [*F*_(5, 434)_ = 9.64, *p* < 0.0001] indicating a difference in learning rate between 2N and Ts65Dn mice (see Figure 2C). Planned comparisons revealed that performance was similar across all four groups during blocks 1 and 2. However, the 2N mice performed significantly better than the Ts65Dn mice during blocks 3–6 (*p* < 0.05). There was no significant effect of maternal diet, nor interactions of diet with either genotype or block. The three-way interaction of genotype, maternal diet, and block was also not significant.

Analysis of the various error types showed significant main effects of genotype on percentage of omissions [*F*_(1, 56)_ = 8.58, *p* < 0.01] and inaccurate responses [*F*_(1, 56)_ = 8.97, *p* < 0.01], but not for premature responses (Table 1). There were no significant effects of maternal diet nor an interaction of diet and genotype for any error type.

#### Study 2 (Young Mice)

In Study 2 (young mice), the analysis of percentage correct for Attention Task 2 revealed a significant main effect of genotype [*F*_(1, 53)_ = 39.99, *p* < 0.0001] with Ts65Dn mice performing more poorly than the 2N mice. There was also a significant main effect of maternal diet [*F*_(1, 53)_ = 4.01, *p* < 0.05]. Although the genotype by maternal diet interaction was only a trend [*F*_(1, 53)_ = 2.43, *p* = 0.1], planned comparisons revealed that within the Ts65Dn mice there was a significant effect of maternal diet (*p* < 0.02) with the Ts+ mice performing better than their non-supplemented counterparts (Figure 2B).

A significant genotype by block interaction was seen for percentage correct [*F*_(5, 253)_ = 15.22, *p* < 0.0001] as well as a significant maternal diet by block interaction [*F*_(5, 253)_ = 4.19, *p* < 0.001]. These effects, illustrated in Figure 2D, showed that choline-supplemented Ts65Dn mice performed better than their non-supplemented counterparts during blocks 3–6, although not as well as 2N mice.

Analysis of error types revealed significant main effects of genotype for percentage of omissions [*F*_(1, 53)_ = 16.84, *p* < 0.001], percentage of inaccurate responses [*F*_(1, 53)_ = 9.75, *p* < 0.01], and percentage of premature responses [*F*_(1, 53)_ = 4.49, *p* < 0.05] (Table 1). There were no significant main effects of maternal diet, nor any significant genotype by maternal diet interactions. For premature response errors, there were significant genotype by block [*F*_(5, 140)_ = 6.81, *p* < 0.0001] and maternal diet by block [*F*_(5, 140)_ = 2.34, *p* < 0.05] interactions. Planned comparisons indicated that the non-supplemented Ts65Dn mice made significantly more premature responses than the supplemented trisomic mice (*p* < 0.05).

### Attention Task 3 (0, 2, or 4 s Variable Pre-cue Delay; 0.8, 1, or 1.4 s Variable Cue Duration)

In both Study 1 and Study 2, pre-cue delay had a significant effect on performance for all groups of mice; percentage correct decreased as the duration of the pre-cue delay increased (*p* < 0.0001). Likewise, cue duration had a significant effect on performance for all subjects; percentage correct decreased as the cue duration decreased (*p* < 0.0001). Pre-cue delay also had significant effects on each error type (premature, inaccurate, omission) for all groups in both studies (*p* < 0.0001). Cue duration had significant effects on inaccurate responses and omissions for all groups in each study (*p* < 0.0001).

#### Study 1 (Aged Mice)

**Figure 3:**
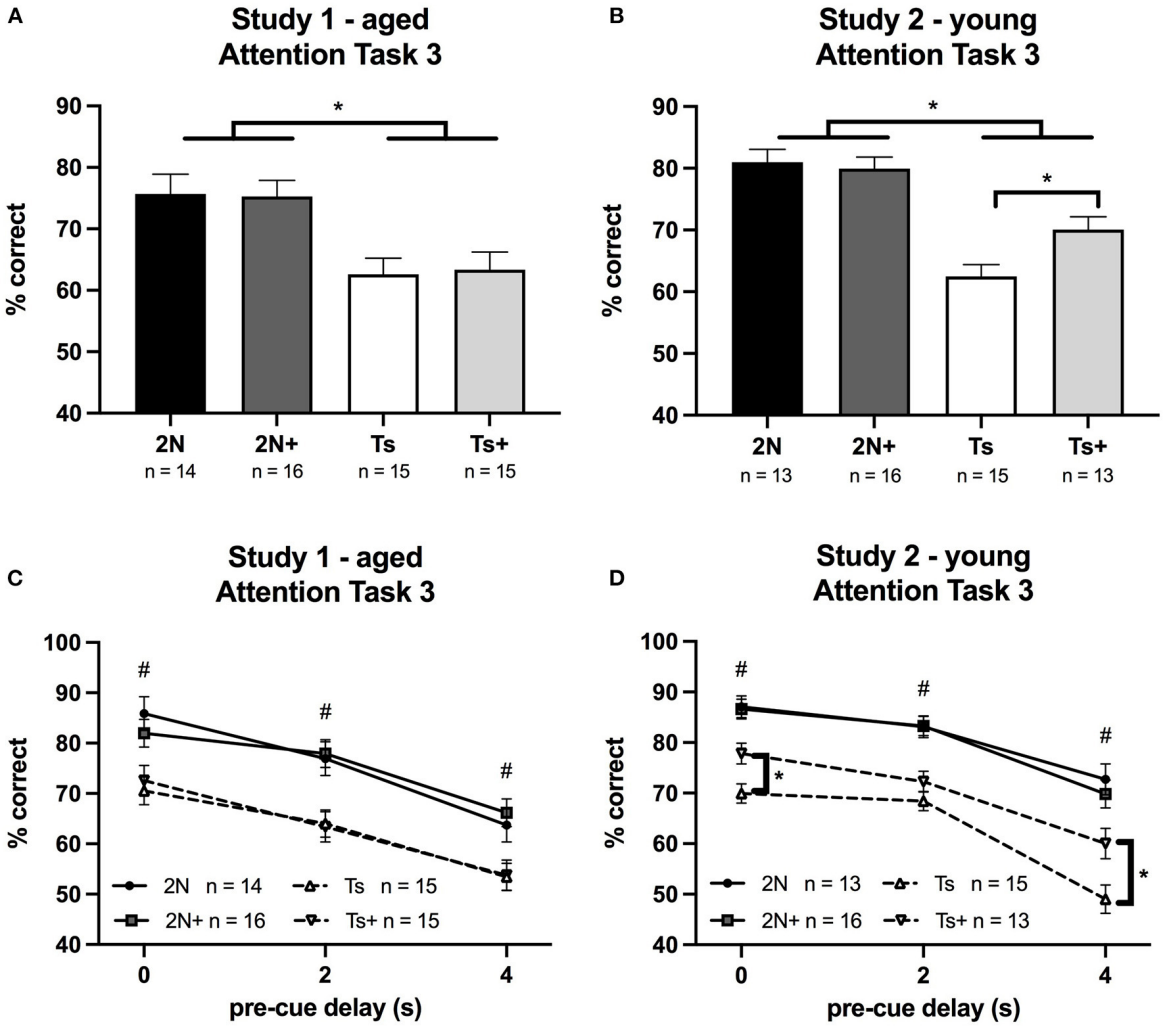
Percentage of correct responses on Attention Task 3 (0.8, 1, or 1.4 s cue duration; 0, 2, or 4 s pre-cue delay) in Study 1 and Study 2. Panels **(A,B)** depict percent correct averaged across the entire task, whereas Panels **(C,D)** depict performance as a function of pre-cue delay. **(A)** In Study 1 (aged mice), a significant effect of genotype was seen, with 2N mice performing better than Ts mice. **(B)** In Study 2 (young mice), a significant effect of genotype was detected as well as a significant genotype by maternal diet interaction. The supplemented trisomic mice performed better than the non-supplemented trisomic mice. **(C)** In Study 1 (aged mice), a significant effect of genotype was detected. **(D)** In Study 2 (young mice), there was a significant genotype by maternal diet by delay interaction. ^#^The 2N and 2N+ groups were both significantly different from each Ts group (*p* < 0.05). *The supplemented trisomic mice performed significantly better than the non-supplemented trisomic mice on trials with a 0 s delay and trials with a 4 s delay (*p* < 0.05).

In Study 1 (aged mice), analysis of percentage correct for Attention Task 3 revealed a significant main effect of genotype [*F*_(1, 56)_ = 19.41, *p* < 0.0001] with Ts65Dn mice performing significantly worse than the 2N mice (Figure 3A). There was no significant effect of maternal diet nor was there a significant genotype by maternal diet interaction. Analysis of errors showed a significant main effect of genotype on the percentage of omissions [*F*_(1, 56)_ = 10.28, *p* < 0.01] (Table 1). There were no significant effects of maternal diet on any error type.

#### Study 2 (Young Mice)

In Study 2 (young mice), the analysis of percentage correct for Attention Task 3 revealed a significant main effect of genotype [*F*_(1, 53)_ = 50.31, *p* < 0.0001] reflecting the fact that the 2N mice outperformed the Ts65Dn mice. In addition, there was a significant genotype by maternal diet interaction [*F*_(1, 53)_ = 4.67, *p* < 0.05]. The choline-supplemented Ts65Dn mice performed significantly better than their non-supplemented counterparts (*p* < 0.01; Figure 3B). The analysis also revealed a significant genotype by maternal diet by delay interaction for percentage correct [*F*_(2, 100)_ = 3.16, *p* = 0.04]. While we observed no effect of MCS in the aged mice (Figure 3C), there was a significant effect of maternal diet in the young Ts65Dn mice (Figure 3D) on trials with a 0 s pre-cue delay (*p* < 0.01) and on trials with a 4 s pre-cue delay (*p* < 0.01), where choline-supplemented Ts65Dn mice performed better than their non-supplemented counterparts. A similar pattern was seen for trials with a 2 s pre-cue delay, but the contrast was not statistically significant.

Analysis of error types indicated significant main effects of genotype on percentage of omissions [*F*_(1, 53)_ = 24.72, *p* < 0.0001], inaccurate responses [*F*_(1, 53)_ = 8.46, *p* < 0.01], and premature responses [*F*_(1, 53)_ = 20.62, *p* < 0.0001] (Table 1); in all cases the trisomic mice made more errors than the 2N. In addition, there was a significant genotype by maternal diet interaction [*F*_(1, 53)_ = 9.92, *p* < 0.01] for percentage of premature responses. The non-supplemented Ts65Dn mice made significantly more premature responses than Ts+ mice (*p* < 0.01).

## Discussion

We reported on the impact of MCS in the Ts65Dn mouse model of DS, assessing learning and attention in two cohorts of mice; one cohort tested at 12 MO (Study 1), and a second at 6 MO (Study 2). In the younger cohort, Ts65Dn offspring born to dams consuming a choline-supplemented diet performed significantly better than their non-supplemented counterparts on each of the three increasingly challenging attention tasks. By contrast, this cognitive benefit of MCS was not observed in the older cohort of Ts65Dn subjects. These findings from young mice replicate and extend the results of our prior study in which testing began at 6 MO (Moon et al., 2010). This earlier study also demonstrated substantial cognitive benefits of MCS for the trisomic offspring. In contrast, the results from the aged mice are similar to our earlier study in which behavioral testing began at 12 MO (Powers et al., 2017). These prior results showed impairment in Ts65Dn mice, but only minor cognitive improvement in choline-supplemented Ts65Dn offspring. Collectively, these four studies indicate that MCS has long-lasting benefits on learning and attention, but the positive effects diminish with age, possibly due to the progression of AD-related neuropathology.

### Nature of the Ts65Dn Impairment and the Benefit Provided by MCS

Important information about the nature of the impairment of the trisomic mice was provided by the types of errors that differentiated them from 2N mice, and also by the pattern of group differences across the different tasks, each with unique cognitive demands. In Task 1, the visual cue was presented at trial onset (as in the prior visual discrimination training task), but the duration of the cue was much shorter than for the training task, which proved to be especially challenging for the Ts65Dn mice. All groups had attained a baseline of at least 80% correct on the training task. However, performance of the 2N mice only modestly declined with the briefer cue. In contrast, the performance of the Ts65Dn mice dropped considerably (Figure 1), primarily due to increased omission errors. In a prior study, videotape analysis revealed that the increased omission errors of the trisomic mice are due to being off-task (facing the side of the chamber opposite to the response ports) (Driscoll et al., 2004). This behavior may be due to cognitive decline in Ts65Dn mice, as they begin to show age-related loss of BFCNs and marked septohippocampal deficits by 6 MO (Granholm et al., 2000; Kelley et al., 2014, 2016).

Task 2 presented the animals with a new rule, which uncovered additional impairments in the trisomic mice. In this task, the visual cue was presented after a variable delay. A response prior to cue onset terminated the trial and was tallied as a premature response error. Since this a difficult task to learn, it initially results in many errors and a dramatic decline in reinforcement rate (relative to the prior task). Previous analysis of videotapes of the animals performing this task revealed that Ts65Dn mice exhibit repetitive jumping specifically after committing an error, indicative of impaired affect regulation; 2N mice do not show this behavior (Driscoll et al., 2004; Moon et al., 2010). Thus, the rate of learning this task reflects emotion regulation, as well as inhibitory control and attentional control. As seen in Figure 2, all groups exhibited poor performance during the first two session blocks (six sessions) on this task due to a high rate of premature responses. By the third session block, the 2N mice showed clear signs of having learned the new rule, performing significantly better than the trisomic mice. This genotype effect was observed in both young and aged mice. This genotype difference in performance during the learning process was due to a higher rate of premature responses, inaccurate responses, and omissions by the Ts65Dn mice reflecting deficiencies in inhibitory control, associative ability, and emotion regulation (Driscoll et al., 2004; Moon et al., 2010; Powers et al., 2017).

Task 3 increases demand on attention and inhibitory control because, in addition to the variability of the pre-cue delay, the cue duration is also variable and very brief on some trials. Ts65Dn mice continued to make more omission-, inaccurate-, and premature response errors than the 2N controls. The most predominant error type in this task in the aged Ts65Dn mice was omissions. However, the trisomic mice also committed significantly more premature and inaccurate responses than the 2N mice, indicative of impaired inhibitory control and impaired attention, respectively.

Several aspects of dysfunction in Ts65Dn mice were ameliorated by increased maternal choline intake. Insight into the nature of the MCS benefit was provided by inspection of the types of errors that exhibited improvement, and also a consideration of the unique demands of each task. In Task 1, the young supplemented trisomic mice displayed improved performance relative to the non-supplemented trisomic mice (Figure 1B) due to a reduction in inaccurate responses. Thus, the benefit of MCS observed in trisomic mice (vs. non-supplemented) on this task likely reflects an improved ability to detect and respond to the now briefer cues (i.e., improved attentional function). On Task 2, the young Ts+ mice were markedly improved relative to their non-supplemented counterparts due to a reduction in premature response errors. When considering task demands, this MCS benefit likely reflects improved inhibitory control, associative ability, and emotional regulation, supporting our earlier findings (Moon et al., 2010). On Task 3, the young Ts+ mice likewise demonstrated improved performance relative to non-supplemented Ts65Dn mice due to a reduction in premature responses indicative of improved inhibitory control in the supplemented mice.

Omission errors contributed to the impaired performance of the Ts65Dn mice in all three tasks, but this error type was unaffected by MCS. Again, based on our videotape analysis of aged Ts65Dn mice (Driscoll et al., 2004), this type of error is associated with the mice being off-task, and not attending to the visual cues, and may reflect cognitive decline associated with AD-like pathological changes. Omission errors were especially predominant in the aged Ts65Dn mice, which did not show a benefit from MCS on these attention tasks. Although the young Ts+ mice demonstrated improved performance (relative to their non-supplemented counterparts), due to reductions in inaccurate and premature response errors, the aged Ts+ mice showed no benefit from MCS. Perhaps, the progression of age-related cholinergic neuropathology overrides the neurological benefits of MCS seen during the younger ages.

In our two prior studies of MCS, we found significant benefits of the added choline for the 2N mice (in addition to the trisomics) although the benefits were only seen at very specific points in the testing, and the effects were small in magnitude (Moon et al., 2010; Powers et al., 2017). In this study, a comparison of the 2N and 2N+ mice revealed trends toward improved performance in the 2N+ mice at identical points in the testing, but the effects were not statistically significant. Specifically, in our previous study of aged mice (Powers et al., 2017), we observed a significant main effect of maternal diet on Attention Task 1, and in fact, a similar trend was seen in this study of aged mice (Figure 1A). In our previous study of young mice, benefits of the MCS were seen only in Attention Task 2 when the animals were first introduced to pre-cue delays: the 2N+ mice were superior to their 2N counterparts during the early learning phase. In this study of young mice, at this identical point in the learning process of Attention Task 2 (block 3), the 2N+ mice tended to perform better than their 2N counterparts (see Figure 2D), but failed to achieve statistical significance. It appears that the benefits of MCS in the 2N mice are subtle, and may not always achieve significance in relatively small samples. In addition, it should be considered that a ceiling effect contributed to the lack of differences between the 2N and 2N+ groups since both the groups performed successfully (~80% correct) on even the most challenging tasks. Prior reviews have noted that the cognitive benefits of MCS for normal rodents are evidenced only under demanding testing conditions (Meck and Williams, 2003; McCann et al., 2006). It was not possible to further increase task difficulty in this study due to the impairment of the trisomic mice.

### Conclusions

The findings of this study suggest that supplementing the maternal diet with extra choline during pregnancy is an extremely promising, high-benefit, low-risk intervention to reduce the cognitive and affective dysfunction in DS. Our translational research using a mouse model of DS has demonstrated that increasing maternal intake of choline during pregnancy and lactation significantly improves attentional function, spatial cognition, and affect regulation in trisomic offspring. These supplemented offspring also exhibit protection of medial septal cholinergic neurons, as well as normalization of hippocampal neurogenesis and expression of genes associated with synaptic plasticity, calcium signaling, and neurodegeneration (Velazquez et al., 2013; Ash et al., 2014; Alldred et al., 2018, 2019). Indeed, there is growing evidence that all pregnant women should increase their intake of choline. Pregnant women in the USA consume on average only ~70% of the Choline Adequate Intake (AI) level recommended by the National Academy of Medicine [Institute of Medicine (US) Standing Committee on the Scientific Evaluation of Dietary Reference Intakes its Panel on Folate, 1998], which is of significant concern in light of the crucial roles that choline plays in fetal neurodevelopment. Moreover, the functional benefit of increasing maternal choline intake, for all pregnancies, is indicated by the results of recent studies that experimentally manipulated choline intake during pregnancy and measured child cognitive outcomes. These studies revealed that higher choline intakes are not only safe and well-tolerated by the mothers, but also improve indices of child cognitive functioning, assessed during infancy (Caudill et al., 2018), toddlerhood (Ross et al., 2016), and 7 years of age (Bahnfleth et al., 2021). There is growing pressure to change obstetric policy so that choline is included in standard prenatal vitamin regimens, which is not currently the case (Caudill et al., 2020). Based on existing animal and human studies, addition of choline to a prenatal vitamin regimen would have population wide benefits likely resulting in improved cognition and affect regulation in all children, as well as providing an early intervention for DS that may protect against AD-associated cognitive decline.

## Data Availability Statement

The raw data supporting the conclusions of this article will be made available by the authors, without undue reservation.

## Ethics Statement

The animal study was reviewed and approved by Institutional Animal Care and Use Committee of Cornell University.

## Author Contributions

BEP conducted all experiments, performed analytic calculations with support from MSS and BJS, and wrote the manuscript with support from BJS and RV. BJS, SDG, and EJM conceived and planned experiments. All authors provided critical feedback to help shape the research, analysis, and manuscript.

## Funding

This work was supported by the National Institute of Child Health and Human Development, grant number HD057564 (to BJS, SDG, and EJM); the National Institute on Aging, grant numbers AG014449 (to SDG and EJM), AG043375 (to SDG and EJM), and AG017617 (to SDG); the Alzheimer's Association, grant number IIRG-12-237253 (to SDG).

## Conflict of Interest

The authors declare that the research was conducted in the absence of any commercial or financial relationships that could be construed as a potential conflict of interest.

## Publisher's Note

All claims expressed in this article are solely those of the authors and do not necessarily represent those of their affiliated organizations, or those of the publisher, the editors and the reviewers. Any product that may be evaluated in this article, or claim that may be made by its manufacturer, is not guaranteed or endorsed by the publisher.
